# A python-based automated pipeline for phylogenetic tree construction, visualization, and comparative statistical evaluation

**DOI:** 10.1242/bio.062482

**Published:** 2026-10-23

**Authors:** Bhavika Gambhava, Janvi Patel, Niyati Buch

**Affiliations:** Department of Computer Engineering, Dharmsinh Desai University, Nadiad, Gujarat 387 001, India

**Keywords:** Phylogenetics, Automated pipeline, Multiple Sequence Alignment, Maximum Likelihood inference, Robinson-Foulds distance, Approximately Unbiased test, Reproducibility

## Abstract

Phylogenetic analysis is a fundamental concept in computational biology, which helps in understanding evolutionary relationships, species divergence, and functional conservation based on biological sequence data. In general, phylogenetic analysis involves a number of sequential steps, such as sequence collection, multiple sequence alignment, model selection, phylogenetic tree construction, and statistical validation, which are usually carried out using different software tools with heterogeneous workflows. In this work, we propose a Python-based phylogenetic analysis pipeline, which integrates a number of important steps in phylogenetic analysis into a unified framework. The proposed system incorporates a number of popular sequence alignment algorithms, such as Multiple Alignment using Fast Fourier Transform (MAFFT), Multiple Sequence Comparison by Log-Expectation (MUSCLE), and ClustalW, as well as different phylogenetic tree reconstruction approaches, such as Neighbor-Joining (NJ), Unweighted Pair Group Method with Arithmetic Mean (UPGMA), Maximum Likelihood (ML), and Maximum Parsimony (MP). In addition, tree visualization, as well as comparative assessment using topological distance measures and statistical hypothesis tests, is performed in the proposed system, which includes Robinson-Foulds (RF), Approximately Unbiased (AU), Kishino-Hasegawa (KH), and Shimodaira-Hasegawa (SH). The results obtained from the analysis of the datasets demonstrate that the combination of the MAFFT-based alignment methods with the ML tree reconstruction strategy results in the generation of trees with high statistical support according to the tests used for the analysis.

## Teaser

**Summary:** An automated, scalable phylogenetic pipeline integrating multiple inference methods-including parsimony-enables reproducible, flexible, and statistically robust evolutionary analysis across diverse biological datasets.

## INTRODUCTION

### Background and the imperative for reproducibility

Phylogenetics provides a framework for reconstructing biological evolution at a molecular level (Woese and Fox, 1977). Apart from this function, phylogenetics has been a key tool in a variety of fields including genomics, molecular epidemiology studies, as well as functional annotations (Philippe et al., 2011). With the exponentially increasing genomic databases along with new sequencing technologies, the demand is urgently increasing to efficiently carry out large-scale, integrated, and comparative studies.

Accurate tree building essentially depends on a workflow that involves data preprocessing, generation of high-quality Multiple Sequence Alignment (MSA), choosing the correct evolutionary model, tree inference, and performing several statistical tests of significance (Felsenstein, 1981; Yang, 2014).

Toolchain fragmentation represents one of the major challenges in high-throughput and reproducible phylogenetic research. While there are efficient alignment algorithms like Multiple Alignment using Fast Fourier Transform (MAFFT) (Katoh and Standley, 2013) and Multiple Sequence Comparison by Log-Expectation (MUSCLE) (Edgar, 2004), and Maximum Likelihood (ML) inference with IQ-TREE (Nguyen et al., 2015), these are separately available as standalone software packages without linking them (Zou et al., 2024). This situation puts serious burdens on researchers for them to deal with different input and output formats, the setting of parameters, and the transition from one software to another. Hence, this situation leads to the lowering of the efficiency level of the analytical workflow and making the workflow process more complicated thus it becomes difficult to reproduce (Guang et al., 2016).

### Defining the research gap and core contribution

The current landscape of available software for phylogenetic reconstruction is highly problematic from a methodological point of view, mainly because of the fragmentation of specialized tools (Guang et al., 2016). Indeed, researchers must apply different, often incompatible, software packages consecutively for the different steps of the phylogenetic reconstruction pipeline (Guang et al., 2016; Dunn et al., 2013; Buch and Gambhava, 2026), from orthologous genes retrieval and MSA generation to tree inference and final statistical validation. The lack of cohesive integration largely hampers scalability and speed in large-scale evolutionary studies and constitutes a known cause of methodological discrepancies, possibly leading to the propagation of errors throughout the analytical workflow (Stamatakis, 2014; Di Tommaso et al., 2017). Additionally, the implicit difficulty in choosing the best methodological pairing, given the distinct theoretical underpinnings and computational features characterizing algorithms such as MAFFT, MUSCLE, ML, and Unweighted Pair Group Method with Arithmetic Mean (UPGMA), too often results in arbitrary choices that reduce the stringency and comparability of results among independent studies (Notredame, 2007).

This research is motivated by the need for an improved computational framework. The main gap that this work tries to fill is the lack of a single, fully automated, Python-based system that can solve the problem of the fragmented toolchain. Crucially, this system must execute a systematic, multi-algorithmic comparative analysis to objectively identify the statistically best-supported phylogenetic hypothesis by comparing competing topologies against the sequence data itself.

The central contribution of this work is to develop and empirically validate a highly reproducible computational framework that achieves end-to-end automation of the complete phylogenetic workflow. This novel system carefully embeds all the requisite sequential stages: flexible handling and acquisition of sequences, generation of comparative alignments [MAFFT (Katoh and Standley, 2013), MUSCLE (Edgar, 2004), ClustalW (Thompson et al., 1994)], several tree-inference mechanisms such as ML (Stamatakis, 2014; Nguyen et al., 2015), Neighbor-Joining (NJ) (Saitou and Nei, 1987; Yang, 2014), UPGMA (Sokal and Michener, 1958), integrated visualization capabilities (Bio.Phylo, ETE3) (Talevich et al., 2012; Huerta-Cepas et al., 2016), and centrally incorporates a multi-metric evaluation framework that stringently adopts both topological distance metrics and statistically robust likelihood-based tests [Approximately Unbiased, AU (Shimodaira, 2002); Kishino-Hasegawa, KH (Shimodaira and Hasegawa, 1999); Shimodaira-Hasegawa, SH (Kishino and Hasegawa, 1989)] to ensure that the final phylogenetic hypothesis obtained is maximally supported and statistically validated.

### Scope and intended usage

While the proposed framework provides a unified and automated environment for phylogenetic analysis, it is important to clearly define the scope of its contribution. The primary objective of this work is not to introduce new phylogenetic algorithms or evolutionary models, but rather to design a comprehensive computational pipeline that systematically integrates widely used bioinformatics tools into a single, reproducible workflow. Traditional phylogenetic studies often require researchers to manually coordinate multiple independent tools across different stages such as data acquisition, alignment, tree inference, and evaluation. This fragmented approach can lead to inconsistencies, reduced reproducibility, and increased computational overhead. The proposed pipeline addresses these challenges by automating the entire workflow, thereby ensuring consistency, traceability, and methodological rigor.

The contribution of this work lies in enhancing workflow efficiency, methodological integration, and comparative evaluation. In addition to supporting multiple alignment strategies and inference methods, the framework incorporates a broader methodological spectrum by including Parsimony-based inference alongside ML and distance-based approaches. This enables a more comprehensive comparison between model-based and non-model-based phylogenetic strategies. Furthermore, the pipeline introduces dynamic data acquisition directly from biological databases, eliminating dependency on pre-curated datasets and allowing flexible dataset generation based on user-defined parameters.

Another important aspect of the framework is its capability to handle large-scale datasets. The pipeline was evaluated on datasets ranging from small collections to larger datasets containing up to 200 species, demonstrating its scalability and stable performance across varying dataset sizes. To support such computational demands, features such as resume functionality and structured execution management have been incorporated. These mechanisms prevent redundant computation by reusing intermediate results, making the system efficient for long-running analyses. Additionally, integration with cloud-based storage (e.g. Google Drive) ensures persistent data management, accessibility, and reproducibility across different execution environments.

Despite these advancements, certain limitations define the scope of the current implementation. While the inclusion of Parsimony extends the diversity of inference methods, the framework does not yet incorporate Bayesian approaches, which are widely used in advanced phylogenetic studies. Moreover, although the system supports moderately large datasets, it is not specifically optimized for ultra-large phylogenomic-scale analyses involving thousands of taxa. Therefore, the current pipeline should be viewed as a scalable and reproducible research framework rather than a replacement for highly specialized high-performance phylogenetic platforms.

The target audience for the proposed pipeline comprises users who are proficient in working in a computational environment and benefit from the reproducibility and automation provided by the framework. This includes graduate students, computational biologists, and bioinformaticians who often perform phylogenetic analysis and wish to test various methodologies for aligning sequences and constructing phylogenetic trees. The framework reduces the burden of coordinating various software tools for the user by incorporating various tools into one framework, allowing the user to replicate the workflow for various datasets.

It must, however, be noted that there are differences between the current framework and other graphical workflow systems, for instance, the Galaxy system (The Galaxy Community et al., 2022). Galaxy provides a web-based system for the construction of bioinformatics workflows using a graphical interface. This system is, therefore, more useful for new users or for individuals without knowledge of programming. However, the present framework additionally provides integrated statistical comparisons of phylogenetic methods within the automation workflow. The proposed pipeline framework provides a command-line-based system implemented using the Python framework, emphasizing the level of automation, as well as the ability to compare phylogenetic strategies. Another important aspect of the system is the evaluation of different alternative phylogenetic topologies using structural and statistical tests. This system is, therefore, more of a supplementary approach to the existing systems.

## COMPUTATIONAL FRAMEWORK AND METHODS

The proposed phylogenetic analysis pipeline is a modular and fully automated framework that is built in Python, with the main aim to increase reproducibility, methodological transparency, and comparative rigor in phylogenetic studies. The general layout of the bioinformatics tool used stresses the smooth integration of the established bioinformatics tools and at the same time keep the systematic evaluation of alternative methodological choices open. Each part of the workflow is done programmatically which means less manual intervention and, therefore, less user-induced variability (Guang et al., 2016; Di Tommaso et al., 2017).

The pipeline is designed to operate across different computational environments such as local workstations and high-performance computing clusters. The main bioinformatics operations are performed using widely adopted open-source libraries that are easy to maintain and validate by the research community. Biopython is used for sequence parsing, format conversion, and basic phylogenetic data handling, while more complex tree comparison operations, such as the calculation of Robinson-Foulds (RF) distance (Robinson and Foulds, 1981), are performed using DendroPy (Moreno et al., 2024). External high-performance tools, such as MAFFT (Katoh and Standley, 2013), MUSCLE (Edgar, 2004), ClustalW (Thompson et al., 1994), and IQ-TREE (Nguyen et al., 2015), are integrated via powerful command-line wrappers that enable consistent execution and standardized input-output handling (Katoh and Standley, 2013; Edgar, 2004; Thompson et al., 1994; Nguyen et al., 2015).

The comparative execution strategy is the distinguishing characteristic of the system. The pipeline for every input dataset will produce 12 candidate phylogenetic hypotheses by the exhaustive evaluation of all possible combinations of three MSA methods and four tree inference approaches. Such an exhaustive pairing strategy makes it possible to directly measure the impact of alignment quality and inference methodology on the resulting phylogenetic topology thus enabling objective method selection based on statistical evidence rather than heuristic preference.

### MSA

MSA is the essential precondition to phylogenetic reconstruction, as the latter is based on the former, i.e. the evolutionary relationships are limited by the accuracy of the identification of the homologous positions in the sequences. Mistakes from the alignment stage are, therefore, the main reasons for putting the tree inference stage into question and, in consequence, wrong topologies and misleading biological interpretations may result. To cope with this problem, the pipeline comprises different alignment methods so that the user is enabled to check whether the effect on the result is alignment dependent.

MAFFT is used as one of the principal alignment tools because of its demonstrated efficiency and accuracy, especially when dealing with a large number of sequences and when there is considerable variation in sequence length. In order to identify the homologous regions, MAFFT utilizes Fast Fourier Transform techniques, thus it can reach a very good alignment quality and, at the same time, keep the method computationally scalable. MAFFT's characteristics illustrate it as the obvious choice for large-scale comparative phylogenomic analysis (Katoh and Standley, 2013).

MUSCLE is selected as another alignment method whose main idea is to use iterative refinement in order to gradually improve alignment quality. MUSCLE is a good compromise between computational efficiency and accuracy, and it is still a method of choice in phylogenetic studies, when alignment has to be both reliable and not too computationally demanding (Edgar, 2004).

ClustalW, a classical progressive alignment algorithm, is included as a reference baseline. While the newest methods are faster and more accurate than ClustalW, the decisions to include it allows one to benchmark against the traditional alignment strategies and compare the results with those of older phylogenetic studies which are based on the progressive alignment paradigms (Thompson et al., 1994).

### Phylogenetic tree inference

Following alignment, phylogenetic reconstruction is performed using multiple inference approaches that differ in their statistical assumptions and computational formulations. This design allows the pipeline to evaluate how inference strategy influences both tree topology and statistical support.

ML inference is employed as the principal character-based method for phylogenetic estimation (Felsenstein, 1981; Yang, 2014). The method looks for a tree topology which maximizes the probability of observing sequence data under a particular model of molecular evolution. ML inference in the current pipeline is achieved via IQ-TREE (Nguyen et al., 2015; Minh et al., 2020), which accounts for automated substitution model selection, fast tree search algorithms, and a variety of integrated statistical testing. IQ-TREE's implementation supports intense likelihood optimization while remaining computationally feasible for datasets of moderate sizes.

In addition to ML, the pipeline incorporates the Maximum Parsimony (MP) method as an alternative character-based inference strategy. Parsimony operates under the principle of minimizing the total number of evolutionary changes required to explain the observed sequence variation. Unlike likelihood-based methods, it does not assume an explicit model of sequence evolution, making it computationally efficient and conceptually straightforward. In this work, Parsimony inference is implemented using the DNAPARS program from the PHYLIP package, with preprocessing steps to ensure compatibility with strict input formats. The inclusion of Parsimony provides an important complementary perspective, enabling comparative evaluation across fundamentally different inference paradigms and enhancing the methodological completeness of the pipeline.

Two distance-based inference methods are also implemented to provide comparative baselines. NJ methods construct phylogenies by progressively joining tax groups in such a way as to minimize the accumulated branch length as defined across pairwise distance matrices (Saitou and Nei, 1987). The method's computational efficiency is what makes it a tool for rapid exploratory analyses; however, it is based on simplified evolutionary assumptions. The UPGMA builds ultrametric trees under the tightly defined assumption of a molecular clock (Sokal and Michener, 1958), that is, all lineages evolve at a constant rate. The method is computationally straightforward, but any violations of this assumption can cause systematic topological distortions; therefore, this method is mainly useful as a reference approach rather than a final inference strategy.

### Tree comparison and statistical evaluation

In order to prioritize objective and statistically sound comparisons of different phylogenetic hypotheses, the pipeline uses a multi-metric evaluation framework to a large extent that topological distance measures are combined with likelihood-based statistical tests. A single metric cannot be overused because the integrated approach also allows for the robust discrimination of the alternative trees.

The Robinson and Foulds distance between the topologically similar trees is measured by how many bipartitions disagree in two phylogenetic trees (Robinson and Foulds, 1981). The metric provides a straightforward and computationally efficient way of assessing the structural differences, but it does not take into account the branch length information. To supplement this limitation, branch length correlation analysis is performed with Pearson correlation coefficients, thus allowing for the evaluation of the consistency of the evolutionary divergence inferred for the corresponding branches.

Verification of the competing tree topologies is facilitated by likelihood-based hypothesis testing through IQ-TREE (Nguyen et al., 2015). The Kishino-Hasegawa (KH) test and the Shimodaira-Hasegawa (SH) test are used here to compare alternative phylogenetic hypotheses generated from the same alignment (Kishino and Hasegawa, 1989; Shimodaira and Hasegawa, 1999). As a solution to the problem of the conservatism of these tests in the case of multiple topologies, the Approximately Unbiased (AU) test is considered as the main criterion for model selection. High AU probabilities signify that a topology is statistically strongly supported (Shimodaira, 2002), while values that are lower than the conventional significance thresholds imply that the topology can be rejected statistically.

## Methodology and implementation

The new, modular, and fully automated phylogenetic pipeline was the basis for the proposed analysis that was conducted. The pipeline was implemented using Python version 3.10 or later. The pipeline was intended to extend reproducibility, flexibility of methods, and statistical rigor at all stages of the phylogenetic workflow, besides the fact that it practically eliminated the manual intervention and user-induced variability.

### System architecture and modularity

The proposed phylogenetic analysis framework is based on a modular architecture and follows a step, driven system, which is shown in the Fig. 1. The pipeline has been automated to the maximum extent and is made in a way that the user has to intervene as little as possible, while at the same time the reproducibility, methodological transparency, and systematic comparison of different phylogenetic strategies are ensured. Each step of the process is a functional module with a clearly defined role, which makes it possible to verify, scale, and extend the process independently in the future.

**Fig. 1.**
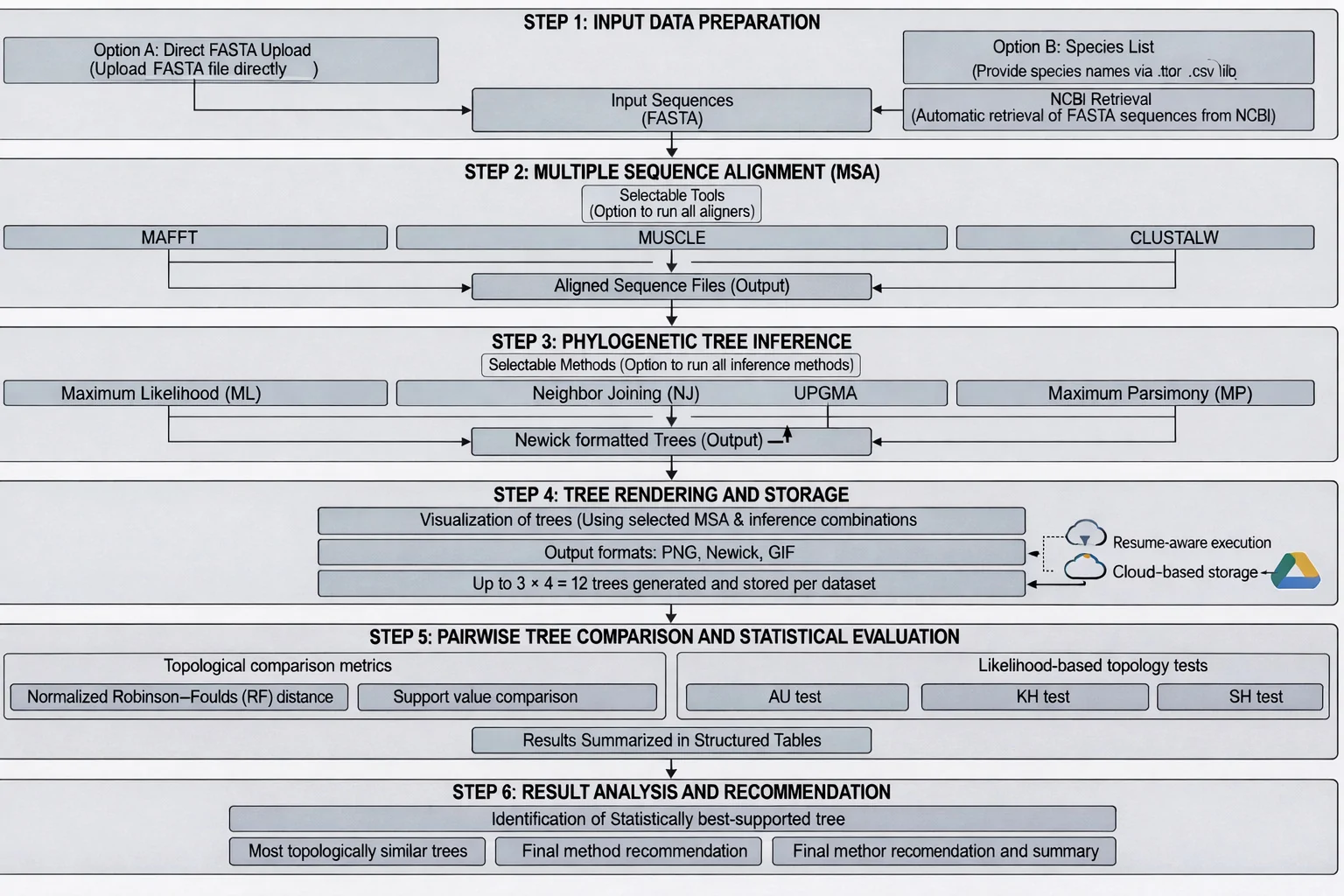
**Overview of** **automatic** **phylogenetic** **analysis** **pipeline with** **step-by-step** **procedure from** **data** **preparation to** **final** **recommendation.**

#### Step 1: Input data preparation

There are two options for the pipeline to accept its biological input. The data input could come from uploading the sequences in FASTA (Fast-All Sequence Format) formatted files (Philippe et al., 2011), or alternatively by uploading the lists of species in text/CSV files. In the second option:

Dynamic dataset generation using user-provided parameters. In the dynamic mode, the user specifies:

- Organism or genus name
- Target gene
- Number of distinct species required

Based on this input, the pipeline automatically queries the NCBI database using programmable interfaces and retrieves sequences corresponding to distinct species. A filtering mechanism ensures that:

- Duplicate sequences are removed
- Only one representative sequence per species is retained
- The longest sequence is selected when multiple entries exist

This feature eliminates the dependency on manually curated datasets and enables scalable and reproducible dataset construction.

#### Step 2: MSA

These sequences are then verified for authenticity and aligned with various sequences using three popular algorithms for sequence alignment, which are MAFFT, MUSCLE, and ClustalW (Thompson et al., 1994; Katoh and Standley, 2013; Edgar, 2004). As per user preference, and/or all three algorithms for sequence alignment are enabled and aligned sequence files are thereby produced for all three with a common output sequence for all three algorithms.

#### Step 3: Inference of phylogenetic tree

The aligned sequence datasets are then proceeded to the inference process where three different approaches are utilized to infer trees: ML with IQ-TREE automated model selection, NJ with distance matrix calculations from alignments, and UPGMA with distance matrix calculations from alignments (Saitou and Nei, 1987; Sokal and Michener, 1958; Felsenstein, 1981), MP using PHYLIP DNAPARS. Every alignment and inference combination produces a unique tree in Newick format.

#### Step 4: Tree rendering and storage

All the trees that have been inferred are shown in the standard way by means of rendering functions, which are contained in the PNG and GIF files. These functions visualize and store Newick-formatted trees. As many as a total of 3×4=12 trees can be generated for each dataset, which are all possible combinations of alignment and inference schemes.

#### Step 5: Pairwise tree comparison and statistical evaluation

The obtained tree topologies are compared on a strict pairwise basis using topological and statistical considerations. Topological assessment takes into account RF distance, differences in branch lengths, and support value agreement. On the other hand, likelihood-based topological tests, for instance, Approximately Unbiased (AU), KH, and SH tests are performed to statistically assess the alternative phylogenetic hypotheses. (Robinson and Foulds, 1981; Kuhner and Felsenstein, 1994; Felsenstein, 1985; Kishino and Hasegawa, 1989; Shimodaira, 2002). The data from all comparison tests are provided in well-organized result tables.

#### Step 6: Results, analysis, and recommendation

In the final stage, the comparative results are combined in order to find the best supported tree hypothesis and the tree with the highest topological consistency. Based on these results, the final report of the optimal strategy for the current dataset regarding the alignment and inference process is made. To conclude, this modular step-by-step approach therefore makes for a fully transparent, reproducible, and therefore evaluable strongly automatized phylogenetic pipeline.

In order to understand the implementation of modularity, the solution architecture in Table 1 highlights the major functional modules of the pipeline according to their main responsibilities. Each function represents an individual task in the pipeline, ranging from input processing and alignment to tree inference, visualization, statistical evaluation, and output. The above-mentioned functions are defined in detail, and the modular approach ensures maintainability, reproducibility, as well as extensibility for future development.

**Table 1.** Core functional modules of the pipeline

Module	Functionality
run_cmd()	Executes external tools, manages subprocess calls, and handles runtime errors.
guess_molecule_from_fasta()	Automatically detects DNA versus protein to select appropriate substitution models.
load_fasta_sequences()	Reads and validates FASTA files; prepares input for alignment.
fetch_sequences_ncbi() / fetch_orthologs()	Retrieves sequences from NCBI/OrthoDB (if user provides species names).
run_blast_search()	Performs BLAST-based ortholog identification when species-only inputs are provided.
perform_alignment_mafft(), perform_alignment_muscle(), perform_alignment_clustalw()	Runs MSA with selectable tools.
infer_nj(), infer_upgma()	Constructs distance-based phylogenetic trees.
infer_ml_iqtree()	Executes ML inference using IQ-TREE (with model selection).
visualize_tree()	Generates static tree images using Matplotlib/Bio.Phylo.
compare_trees()	Computes RF distance and branch-length correlation between inferred trees.
run_likelihood_tests()	Performs AU, SH, and KH model-fit tests for statistical evaluation.
analyze_results()	Summarizes all results, identifies the best-fit tree, and prepares the final report.
export_outputs()	Saves alignments, trees, plots, and comparison tables in a structured output directory.

The above table illustrates how the modular structure is later specialized into concrete functions, each responsible for a particular part of the phylogenetic analysis. By organizing the pipeline in this fashion, the system provides not only automated re-execution but also a transparent framework that can easily be modified to include different analytical methods. In addition, the modular framework permits scientists to determine which step is correlated with any given output or metric, which helps in troubleshooting and methodological evaluation.

### Integrated algorithms for comparative analysis

In order to enable a systematic comparison of the different methodological approaches, this pipeline applies all combinations of three MSA and four tree inference methods. This experiment setup mentioned above will result in twelve different possible phylogenetic trees for every data set. In this manner, it will be feasible to make an objective appraisal of the effect of alignment as well as inference approaches to the result of the phylogenetic analysis.

#### MSA

Alignment strategy affects the following phylogenetic analysis; hence, three popular algorithms in the field of the MSA problem were combined. MAFFT is preferred because of the efficiency and accuracy of the algorithm and the ability to handle multiple sequences of large size (Katoh and Standley, 2013). MUSCLE is incorporated because of the balance of efficiency and alignment quality (Edgar, 2004). Additionally, the simplest progressive alignment strategy in the literature-namely, the ClustalW (Thompson et al., 1994) algorithm is used as the fixed solution in order to be comparable with the classical phylogenetic analysis pipelines. While working with the ClustalW alignment algorithm, the distance between a pair of sequences is measured by the relation:

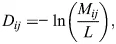
where *M*_*ij*_ denotes the number of matched positions and *L* represents the alignment length.

#### Tree inference methods

To reconstruct phylogenetic trees, both character-based and distance-based methods were employed. Among the character-based approaches, ML and MP were used. ML searches for the tree topology that maximizes the likelihood of the observed sequence data under a specified model of evolution (Stamatakis, 2014; Nguyen et al., 2015). The analyses were performed using IQ-TREE (Nguyen et al., 2015), which provides built-in model selection and efficient likelihood optimization. In contrast, MP identifies the tree requiring the minimum number of evolutionary changes without assuming an explicit model of sequence evolution. MP analyses were carried out using the DNAPARS program from the PHYLIP package with appropriate preprocessing and automation. To complement these character-based methods, two distance-based approaches were also employed. Neighbor Joining reconstructs trees from pairwise distance matrices by iteratively minimizing total tree length (Saitou and Nei, 1987; Yang, 2014), whereas the UPGMA constructs trees under the assumption of an ultrametric tree and a strict molecular clock (Felsenstein, 1981; Sokal and Michener, 1958).

### Tree comparison and evaluation framework

In order to find the best phylogenetic hypothesis out of the competing ones objectively, the proposed pipeline uses a tree comparison and evaluation framework. This framework allows the simultaneous evaluation of the structural similarity and statistical significance. This helps to select the best tree among the competing ones that are created from each dataset. A conceptual example is presented in Fig. 2.

**Fig. 2.**
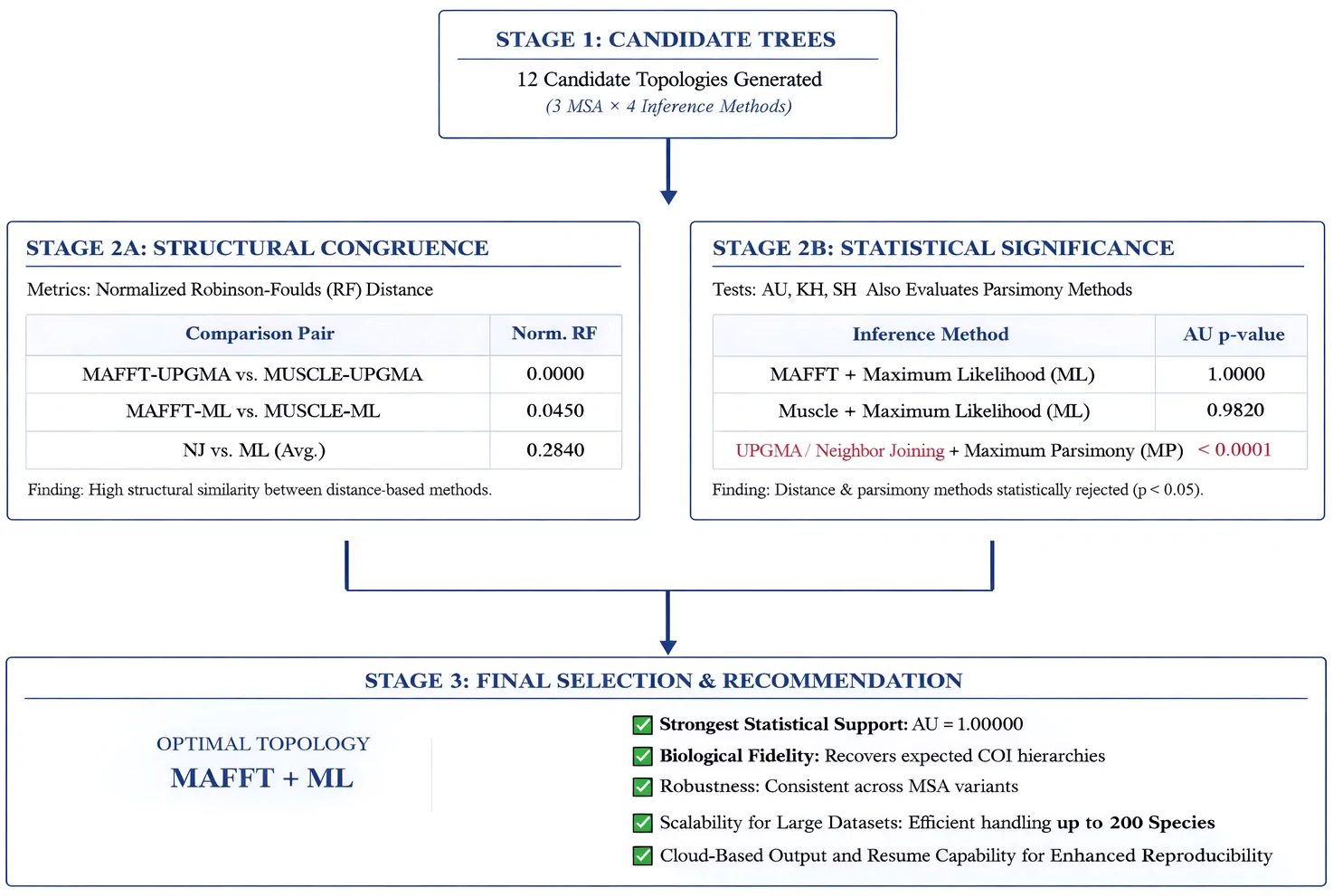
Multi-stage tree comparison and evaluation framework illustrating candidate tree generation, structural congruence assessment, statistical hypothesis testing, and final topology selection using an example dataset.

#### Stage 1: Candidate tree generation

In the case of every dataset, the pipeline checks all possible combinations of the MSA algorithms and phylogenetic inference approaches. This leads to twelve possible tree topologies being obtained depending on the assumptions proposed by the algorithms. These trees serve as the point for comparison.

#### Stage 2A: Structural congruence analysis

In the initial assessment stage, the focus is on the analysis of the topological consistency of the candidate tree solutions using the RF normalized distance metric (Robinson and Foulds, 1981). In this case, the distance metric determines the level of inconsistent bipartition information within the candidate tree solutions, with lower values indicating a higher degree of tree similarity.

As shown in Fig. 2, the distance-based algorithms UPGMA and NJ have strong topological agreement, and the RF-distances between the trees from different alignments are close to zero. Slightly higher RF differences are found between trees from distance-based and character-based (ML and MP) methods, showing the effect of tree estimation on tree topology. Structural analyses offer an explanation of the variability introduced by the methods without invoking early statistical rejection.

#### Stage 2B: Statistical significance testing

While structural similarity measures topology similarity, statistical support is not implied by similarity measures. Consequently, the second level of structural similarity evaluation entails statistical hypothesis testing through likelihood to ascertain if there are any statistically significant differences among topology hypotheses.

In the IQ-TREE model (Nguyen et al., 2015), the pipeline performs KH tests, SH tests, and AU tests (Kishino and Hasegawa, 1989; Shimodaira, 2002) for tree topology comparisons.

In the example from Fig. 2, ML trees under both alignment programs of MAFFT and MUSCLE reveal strong support values under the AU criterion, contrary to the distance methods that do not receive support at any regular alpha values despite the presence of topological similarity. It is evident that the topological and statistical evaluations should be considered in conjunction.

#### Stage 3: Final topology selection and recommendation

The last step in selection combines evidence derived from structural congruence and statistical testing. The model that is statistically supported, biologically plausible, and robust against various alignment strategies will be preferred as the best phylogenetic tree.

In this specific case, the MAFFT+ML tree returned the highest probability under the AU criterion; hence, it is indicated as the best tree topology supported. This decision-making process is important because it removes any dependence on preference for methods to serve as the basis for final suggestions.

### Experimental setup and datasets

#### Development environment and software tools

The pipeline was developed and executed in a Python 3.10+ environment. The necessary libraries were Biopython (Cock et al., 2009) for sequence manipulations and formats, DendroPy for the calculation of the RF distance, and SciPy for the statistical analysis of correlations. The local installations of the command line programs were managed by optimized command line interfaces and consisted of: MAFFT (Katoh and Standley, 2013), MUSCLE (Edgar, 2004), ClustalW (Thompson et al., 1994), and IQ-TREE (Nguyen et al., 2015) for ML tree inference and validation. The automatic detection of a nucleic acid or protein sequence and the choice of the respective evolutionary model were performed by guess_molecule_from_fasta().

#### Dataset introduction

The automated phylogenetic pipeline was validated for different biological groups of ortholog pairs with varying evolutionary distances, using various genetic markers, and covering different molecular complexities. All these sets of biological data are generated from openly available sources, including GenBank at NCBI (National Center for Biotechnology Information) (Sayers et al., 2021) and BLAST (Basic Local Alignment Search Tool) databases (Camacho et al., 2009), thus making these biological inferences as relevant and representative as possible. The exact choice of biological data, however, is based on the facility to check the pipeline's robustness for various molecular complexities.

- Dataset 1: Vertebrate COI (cytochrome oxidase subunit I). This dataset comprises mitochondrial COI sequences from representative vertebrates, including mammals such as *Homo sapiens*, *Pan troglodytes*, and *Mus musculus*. COI is a widely employed marker for resolving deep phylogenetic relationships within the animal kingdom and is particularly effective at distinguishing among vertebrate lineages (Pentinsaari et al., 2016; Satoh et al., 2016). This dataset comprised 11 sequences with lengths ranging from 132 bp to 803 bp (average length 571.9 bp).
- Dataset 2: Bacterial 16S rRNA. These datasets include 16S ribosomal RNA (rRNA) gene sequences of common Enterobacteria species such as *Escherichia coli* and *Shigella flexneri*. In bacterial identification and phyloclassification, 16S ribosomal RNA gene sequences have been widely accepted as molecular signatures. These genes help test the algorithm's ability to correctly discern the phylogenetic relationship in the bacterial kingdom (Woese and Fox, 1977; Sokal and Michener, 1958). This dataset comprised nine sequences with lengths ranging from 490 bp to 1479 bp (average length 1117.1 bp).
- Dataset 3: Plant rbcL (ribulose-1,5-bisphosphate carboxylase/oxygenase large subunit). Chloroplast rbcL sequences from genera such as *Brassica* and *Solanum* were included to evaluate the pipeline's capacity to reconstruct plant evolutionary relationships. The rbcL gene is a gene of choice in phylogenetic analysis in plants because it has a mid-rate of evolution and is also highly conserved among different species (Hilu et al., 2003; Kress and Erickson, 2007). This dataset comprised nine sequences with lengths ranging from 762 bp to 2433 bp
- Dataset 4: Insect COI (Hymenoptera). The mitochondrial dataset of gene COI in this case concentrates on species of insects close to each other in relation to the Hymenoptera order of insects, species of which include *Temnothorax*. It enables assessment of the pipeline's resolution power in distinguishing fine-scale speciation events among closely related taxa (Prebus, 2017). COI sequences were retrieved from NCBI, and the longest available sequence for each taxon was selected for analysis. A total of 40 taxa were included in this dataset, and larger datasets comprising approximately 50, 100, 150, and 200 species were additionally used to evaluate pipeline scalability.
- Dataset 5: Insect Hsc70-4 (heat shock cognate 70-4). Nuclear Hsc70-4 sequences from the *Bombus* genus were included to test the pipeline's performance on nuclear markers with potentially different evolutionary dynamics compared to mitochondrial and ribosomal genes. This dataset challenges the pipeline to accurately reconstruct phylogenies from genes with variable substitution rates (Nguyen et al., 2016). Hsc70-4 sequences were obtained from NCBI, and the longest available sequence for each species was used in the analysis. A total of 25 sequences were included in this dataset.

The use of these five datasets enables assessment of the pipeline performance on different aspects, including gene types (mitochondrial, nuclear, ribosomal, and chloroplast), sequence divergence, and taxonomic groups. The scalability experiments further evaluated computational efficiency, stability, and the effectiveness of optimization features such as resume-based execution and structured storage under increasing data complexity

### Automated execution flow

Sequences are then autonomously aligned with MAFFT (Katoh and Standley, 2013), MUSCLE (Edgar, 2004), and ClustalW (Thompson et al., 1994) to yield three different alignment formats. For each alignment format, phylogenetic trees are reconstructed with NJ (Saitou and Nei, 1987; Yang, 2014), UPGMA (Sokal and Michener, 1958; Felsenstein, 1981), and ML inference (Stamatakis, 2014; Nguyen et al., 2015), giving 12 topological models, and additionally, a Parsimony-based approach implemented via DNAPARS, thereby extending the inference framework beyond model-based methods. Each alignment–inference combination produces a distinct tree topology, which is stored in a structured format. To improve computational efficiency, the pipeline incorporates a resume-aware execution mechanism, allowing previously computed results to be reused and avoiding redundant processing – particularly beneficial for large datasets. Such trees are then analyzed using topological methods and statistics, such as RF topological distance, branch length correlation, and statistical testing of hypotheses. Finally, all quantified data are integrated and interpreted to yield a statistically supported recommendation of the optimum phylogenetic hypothesis.

### Code and data availability

To ensure full reproducibility and transparency, both the implementation of the proposed phylogenetic pipeline and the datasets used in this study have been made publicly available. The repository includes the complete source code, execution scripts, configuration files, and sample outputs, along with curated datasets used for experimental validation.

The datasets consist of representative biological sequences retrieved from publicly available databases such as NCBI and include processed FASTA files for all evaluated cases. Additionally, instructions for regenerating datasets using the dynamic data acquisition module are also provided.

The complete resource can be accessed at: https://github.com/niyatibuch/automated-phylogenetic-pipeline.

## RESULTS AND DISCUSSION

This part is a consolidated analysis of the quantitative results along with their biological and methodological implications. These results came from the evaluation of the automated phylogenetic pipeline. Five biologically diverse sets of orthologs were each subjected to all the 12 combinations of MSA and tree inference strategies. The resulting phylogenetic hypotheses were tested with likelihood-based statistical tests such as the AU (Shimodaira and Hasegawa, 1999), KH (Kishino and Hasegawa, 1989), and SH (Shimodaira, 2002) tests along with topological comparison by the RF distance.

In addition to standard validation datasets, the pipeline was also tested on large-scale datasets ranging from 50 to 200 species, enabling assessment of scalability, computational stability, and performance under increasing data complexity. The inclusion of resume functionality ensured efficient handling of such large experiments.

### Statistical superiority of likelihood-based inference

Comparing all the datasets that were subject to evaluation, the same clear and unambiguous pattern was found. In each instance the combination of MAFFT-based alignment and ML inference was able to attain the highest statistical support, with AU test probabilities of 1.0000 for the best-supported topology. This single result is evidence for the robustness and stability of likelihood-based inference when combined with high-quality sequence alignment.

The inclusion of the Parsimony method provided an additional comparative perspective, revealing that while Parsimony can produce biologically reasonable trees, it lacks the statistical framework necessary for hypothesis testing and therefore does not consistently match the confidence levels achieved by ML.

On the other hand, phylogenetic trees constructed using distance-based methods, particularly NJ and the UPGMA, frequently showed lower statistical support when compared with the best-supported ML topology. These methods often produced AU probabilities close to zero, indicating statistically weaker support. This observation suggests limitations of distance-based approaches in fully capturing evolutionary processes such as rate heterogeneity and substitution bias.

### Topological consistency

For evaluating the degree of agreement regarding the tree structure among the competing phylogenetic topologies, the normalized RF (Robinson and Foulds, 1981) distance was used as a topological similarity measure. The minimum values of RF distances between the closest non-optimal topologies for each dataset are shown in Table 2.

**Table 2.** Topological stability and method sensitivity across datasets

Ortholog/Dataset	Taxonomic scope	Most similar tree pair	Min. norm. RF	Max. norm. RF	Primary source of variation
Vertebrate COI	Mammals	MAFFT+UPGMA versus MUSCLE+UPGMA	0.125	0.686	Inference method (ML versus distance)
Bacterial 16S rRNA	Enterobacteriaceae	MAFFT+NJ versus MUSCLE+NJ	0.000	0.000	None observed
Plant rbcL	Angiosperms	MAFFT+NJ versus MUSCLE+NJ	0.000	0.000	None observed
Insect COI (*Temnothorax*)	Ant genus-level	MAFFT+UPGMA versus MUSCLE+UPGMA	0.000	0.771	Inference method
Insect Hsc70-4 (*Bombus*)	Hymenoptera	MAFFT+NJ versus CLUSTALW+NJ	0.048	0.657	Alignment and inference interaction

The values of RF distances range from 0.0000 to 0.1250, showing that a number of suboptimal trees exhibit a high degree of topological similarity. A perfect topological fit between a number of distance-based topological trees was observed for highly conserved regions like bacterial 16S rRNA and plant ribulose-1,5-bisphosphate carboxylase large subunit genes, as supported by a RF distance value of 0.0000. This suggests that if the sequences are highly conserved, high topological signal may dominate over topological differences among methods.

However, topological variation did increase with greater complexity or rapid evolution among the data sets, especially for the vertebrate cytochrome c oxidase subunit I marker, which had the largest RF topological distance. This again reflects the relevance of tree selection to methodological choice when it has significant topological implications.

Near-zero minimum RF values across datasets indicate that a common underlying topology is recovered irrespective of alignment method due to strong phylogenetic signal. Maximum RF distances show observed increases, associated mainly with the use of distance-based inference methods, especially for protein-coding genes, whereas ribosomal and chloroplast markers remain invariant in topology. Such a pattern thus indicates that the topological differences arise from methodological sensitivity rather than instability in the data, confirming the robustness of the proposed pipeline.

### Integrated comparative performance across datasets

A consolidated overview of the optimal methodological configurations and their corresponding quantitative metrics across all datasets is provided in Table 3.

**Table 3.** Summary of likelihood-based test outcomes across datasets

Ortholog/dataset	Sequence type	Dominant method	AU support	KH/SH test outcome
Vertebrate COI	Mitochondrial DNA	MAFFT+ML	1.000	Weakly informative
Bacterial 16S rRNA	Ribosomal RNA	MAFFT+ML	1.000	Non-discriminatory
Plant rbcL	Chloroplast DNA	MAFFT+ML	1.000	Non-discriminatory
Insect COI (*Temnothorax*)	Mitochondrial DNA	MAFFT+ML	1.000	Weakly informative
Insect Hsc70-4 (*Bombus*)	Nuclear protein-coding gene	MAFFT+ML	1.000	Moderately informative

While Parsimony and distance-based methods contributed to broader methodological comparison, they primarily served as complementary approaches rather than optimal solutions.

The inclusion of multiple inference paradigms, combined with large-scale dataset validation, highlights the pipeline's capability to systematically evaluate phylogenetic strategies under diverse biological and computational conditions.

Table 3 summarizes the statistically optimal phylogenetic inference outcomes on all validation datasets. In each of them, the maximum likelihood tree obtained with MAFFT alignment consistently shows the highest support scores under the AU test, revealing a preeminent model of evolution. The absence of discriminatory power of KH and SH tests makes sense due to the presence of high phylogenetic signal, under which other trees are systematically and overwhelmingly rejected. The slight variation of protein-coding genes detected with minimal genetic changes does not impact the statistical preeminence of model-based inference to render confidence to the process.

### Biological interpretation and methodological implications

The phylogenetic trees with the best statistical support obtained through the pipeline reflected evolutionary relationships that have been confirmed by previous studies for all taxa examined. In this case, mitochondrial cytochrome c oxidase subunit I markers were not only able to resolve the major vertebrate divergences but also the fine-scale relationships within the genera of insects. On the other hand, conserved ribosomal and chloroplast markers gave the most stable topologies that are in line with the phylogenies known. These findings serve as an indication of the biological relevance of the trees that were derived and they also prove the pipeline's ability to work with datasets that cover different evolutionary timescales.

The continuous selection of ML (Stamatakis, 2014; Nguyen et al., 2015) inference most clearly indicates the importance of explicitly modeling evolutionary processes. When substitution models are used and rate heterogeneity is taken into account, likelihood-based methods offer a statistically strong framework that is not outperformed by distance-based methods, especially in datasets with complex evolutionary dynamics.

### Strengths, reproducibility, and limitations

Functionality wise, the pipeline can be classified as an end-to-end automated framework that combines very easily the steps of sequence acquisition, alignment, inference, visualization, and statistical validation within a reproducible workflow. Its promise to work only with open-source tools and the standard output format means that the results are transparent, and anyone can easily reproduce their analysis. Additionally, the design also enables the installation of the different levels of high-performance computing, hence the scalability required by larger datasets. The introduction of dynamic input handling, Parsimony-based inference, and large-scale dataset support significantly extends its analytical capabilities. The implementation of resume functionality improves computational efficiency by avoiding redundant processing, while cloud-based storage integration ensures persistent and reproducible outputs.

However, some boundaries are still left. Inference methods using likelihood functions, as well as statistical tests related to them, require a considerable amount of computation, hence might affect the performance rate in massive data of phylogenomic sequences. Moreover, to date, implemented strategies are restricted to NJ methods (Saitou and Nei, 1987; Yang, 2014), UPGMA (Sokal and Michener, 1958; Felsenstein, 1981), and Phylogenetic Likelihood (Stamatakis, 2014; Nguyen et al., 2015). Lastly, this pipeline targets nucleotide and protein sequences with the development of this pipeline to different data types being an important advancement.

### Conclusion

In this paper, we describe a completely automated and reproducible framework for phylogenetic analysis that combines various steps of sequence analysis into a single framework. The novelty of this work is the integration and automation of various steps of phylogenetic analysis and their systematic comparison for different alignments and methods of tree inference.

The effectiveness of the proposed framework is illustrated through an experimental evaluation on various orthologous datasets, which shows how different combinations of methods can affect the outcome of phylogenetic analysis and the degree of statistical support for the inferred trees. For instance, in the datasets used for this evaluation, combinations of MAFFT-based alignments and ML tree inference methods showed good statistical support for the inferred trees under likelihood-based tests.

The novelty of this work is not to claim the superiority of any specific combination of methods but to demonstrate the effectiveness of the proposed framework for reproducible and systematic comparison of various methods of phylogenetic analysis. The framework is designed to be used for analyzing large datasets and to be expanded to include other methods of phylogenetic analysis in the future.

The proposed framework should be viewed primarily as an integrated and reproducible workflow framework for comparative phylogenetic analysis rather than a novel phylogenetic inference methodology.

### Future work

In future work, the proposed framework can be extended into a more intelligent and adaptive system for automated phylogenetic analysis by integrating data-driven and learning-based approaches. One key direction involves incorporating intelligent decision-making mechanisms to automatically select optimal alignment strategies, inference methods, and model parameters based on dataset characteristics, thereby reducing dependency on manual configuration.

Further improvements may include optimization of computational performance through parallel processing and resource-aware scheduling, particularly for large-scale datasets exceeding hundreds of species. The integration of advanced inference paradigms, such as Bayesian methods and enhanced Parsimony models, can further strengthen the analytical depth of the pipeline.

Additionally, the framework can be extended to support more complex biological data types, including genomic, transcriptomic, and structural datasets, broadening its applicability in comparative genomics. The development of interactive and web-based visualization interfaces (e.g. API-based integration with advanced tree visualization platforms) can further improve interpretability and usability.

Importantly, all future enhancements will be designed as modular extensions to the existing architecture, ensuring that the core principles of reproducibility, scalability, and flexibility are preserved. This design makes it possible to incorporate new methods and analytical components without interfering with the established workflows. Therefore, the framework provides a stable and extensible foundation for developing and managing increasingly sophisticated phylogenetic analysis pipelines as computational and methodological requirements evolve.

## Supplementary Material


