# Supplementary material for: A python-based automated pipeline for phylogenetic tree construction, visualization, and comparative statistical evaluation
Source: Biol Open. 2026 Oct 23;15(10):bio062482. doi: 10.1242/bio.062482 (PMC13730138; doi:10.1242/bio.062482)
Supplement: Supplementary file 1 [file biolopen-15-062482_review_history.pdf]

A Python-Based Automated Pipeline for Phylogenetic Tree Construction, Visualization, and Comparative Statistical Evaluation

Janvi Patel, Niyati Buch and Bhavika Gambhava
10.1242/bio.062482

Editor: Marie Monniaux

Review timeline
Original submission: 20 January 2026
Editorial decision: 29 January 2026
First revision received: 12 April 2026
Editorial decision: 7 June 2026
Second revision received: 21 June 2026
Accepted: 30 June 2026

Original submission

First decision letter

MS ID#: bio.062482

MS Title: A Python-Based Automated Pipeline for Phylogenetic Tree Construction, Visualization, and Comparative Statistical Evaluation

Authors: Bhavika Gambhava, Janvi Patel and Niyati Buch

I have now reached a decision on the above manuscript.

The reviewer reports are shown at the bottom of this email.

As you will see, the reviewers raised a number of substantial criticisms that prevent me from accepting the paper at this stage.

They suggest, however, that a revised version might prove acceptable, if you can address their concerns. If you think that you can deal satisfactorily with the criticisms on revision, I would be pleased to see a revised manuscript. We would then return it to the reviewers.

Specifically, both reviewers raised concerns about the novelty of the proposed pipeline, and its target audience. Integrating e.g. Bayesian approaches would increase the novelty but represents substantial work. Alternatively, clarifying the scope of the pipeline and toning down the claims of the manuscript would be required for the manuscript to be considered for publication. The concern of reviewer 1 on validation of the pipeline is also to be considered: is it feasible to validate the pipeline with larger datasets? Please also provide the alignments as requested by reviewer 2, and answer to all other comments from both reviewers.

At this stage, we also ask you to ensure your manuscript complies with our formatting guidelines. Provided you are able to fully address the referees' comments, we are positive about publication of your paper (we accept over 95% of revision submissions) and therefore hope you won't mind any extra work involved in reformatting your manuscript at this point.

© 2026. Published by The Company of Biologists under the terms of the Creative Commons Attribution License (https://creativecommons.org/licenses/by/4.0/).

Please upload both a 'clean' version of your Word file, along with a highlighted version clearly showing where you have made changes in the revised manuscript. Please avoid using 'Track changes' in Word files as these are lost in PDF conversion.

I should be grateful if you would also provide a point-by-point response detailing how you have dealt with the points raised by the reviewers in the 'Response to Reviewers' box. Please attend to all of the reviewers' comments. If you do not agree with any of their criticisms or suggestions please explain clearly why this is so.

Reviewer 1

Comments for the author

This manuscript presents a Python-based pipeline that integrates multiple stages of phylogenetic analysis, including sequence alignment, tree inference, visualization, and statistical comparison of phylogenetic trees. It is interesting to see an effort that aims to implement an end-to-end phylogenetic workflow entirely within Python, rather than relying on a collection of separate software packages. The goal of providing an integrated and potentially reproducible workflow would be of interest to researchers in the field, and the manuscript has merit. However, in its current (revised) form, I still have several conceptual, methodological, and validation-related concerns that may limit confidence in the generality of the proposed approach.

Overall, the manuscript reads more as a software integration effort than as a methodological advance in phylogenetic inference. I suggest that this point be explicitly acknowledged and clearly stated in the abstract and/or conclusions. Over the past two decades, phylogenetic research has advanced substantially, and the most widely used and reliable approaches for tree inference now include Maximum Likelihood, Bayesian inference, and parsimony-based methods (particularly for morphological character matrices). Other approaches implemented in the pipeline. From a scientific perspective, accuracy and robustness of phylogenetic inference are typically prioritized over convenience of implementation, particularly for analyses addressing large scale and/or deep-time evolutionary questions, where results can vary substantially among methods. Is it possible to integrate Bayesian and Most Parsimony methods into the pipeline? I understand that this could be separate works, but it is definitely more helpful than others.

While the proposed pipeline provides a more streamlined and convenient workflow from data processing through tree inference and downstream analyses, which is potentially useful for certain applications, this convenience alone may not be a core scientific advance. Clarifying the intended scope and limitations of the pipeline would help align the manuscript's claims with its actual contributions.

Regarding validation, the initial evaluation based on only five orthologous sequences was insufficient to support claims of general applicability and reproducibility. Although the revised manuscript has made some improvements in this respect, the validation remains relatively limited, and broader benchmarking on larger and more diverse datasets would further strengthen the study.

Finally, I recommend removing or rephrasing the term "methodological advantage" throughout the manuscript, as this claim is not sufficiently supported from a scientific standpoint. A more cautious description emphasizing integration, usability, or proof-of-concept implementation would be more appropriate.

Typo in the reference list:
[9] author names
[21] Journal name abbreviation "Mol Biol Evol."
[31], "Muller"

In addition, I did not find the reference [17] Wu H., Li Z., Zhang Y., Chen X. (2024). Global patterns of COI gene diversity across vertebrates. Genes, 15, 1037. Please check it.

Reviewer 2

© 2026. Published by The Company of Biologists under the terms of the Creative Commons Attribution License (https://creativecommons.org/licenses/by/4.0/).

Comments for the author

The reason why I selected no for sufficient raw data is that the supplement, while including all the commented code, does not include the alignments themselves that are tested in Tables 2 and 3. I also selected no for reproducibility for this reason, but including the alignment would change both to a yes.

I selected yes for both scholarship questions, but neither question is entirely relevant to this method. I also selected yes for the question of whether other relevant experiments could be performed that were not included (note: this question only appears in the instructions to authors, and not in the drop down menu - I am not sure why the questions are different). This is because there are many other phylogenetic reconstruction algorithms and tree comparison statistics that exist. However, I do not think it is necessary to include them in such a basic pipeline.

I would like to see mention of who is the target audience for this pipeline. It functions similarly to Galaxy, except that I am not sure that Galaxy includes the topology comparisons/tests (which are clever to include). However, for new users unfamiliar with bioinformatics, Galaxy has the advantage of being drag-and-drop, while the pipeline described here requires command line and installation of a Python environment.

Can this pipeline be used on concatenated alignments of multiple genes?

Minor comments:
- Page 3, line 35, column 2: "Step 4: Tree Rendering and Storage"
- Page 3, line 55, column 2: "Results, Analysis, and Recommendation"
- Page 4, line 41-42, column 1: I don't understand what this is trying to say
- Page 6, line 62, column 1: Please italicize species names
- Page 8, line 49-50, column 1: This sentence does not completely make sense, please reword

Reviewer's Responses to Questions

Experimental quality
Does each figure have the proper controls?
If 'No', please indicate reasons in Comments for Author box below.
Reviewer #1:
- Yes

Reviewer #2:
- Yes

Were the data analyzed using appropriate statistical tests?
If 'No', please indicate reasons in Comments for Author box below.
Reviewer #1:
- Yes

Reviewer #2:
- Yes

Reproducibility
Were experiments performed using adequate number of biological replicates?
If 'No', please indicate reasons in Comments for Author box below.
Reviewer #1:
- Yes

Reviewer #2:
- Yes

© 2026. Published by The Company of Biologists under the terms of the Creative Commons Attribution License (https://creativecommons.org/licenses/by/4.0/).

Does the methods section provide sufficient detail to permit reproducibility?
If 'No', please indicate reasons in Comments for Author box below.
Reviewer #1:
- Yes

Reviewer #2:
- No

Completeness
Are the manuscript's conclusions supported by the data?
If 'No', please indicate reasons in Comments for Author box below.
Reviewer #1:
- No

Reviewer #2:
- Yes

Scholarship
Do the authors cite and discuss the merits of data that would argue for and against their conclusion?
If 'No', please indicate reasons in Comments for Author box below.
Reviewer #1:
- Yes

Reviewer #2:
- Yes

Does the manuscript title & abstract accurately reflect the contents of the manuscript, without hyperbole?
If 'No', please indicate reasons in Comments for Author box below.
Reviewer #1:
- No

Reviewer #2:
- Yes

First revision

Author response to reviewers' comments

Response to Reviewer 1

Comment 1: Paper reads as software integration rather than methodological advance
Response:
We agree with the reviewer. The primary contribution of this work is **integration, automation, and comparative evaluation**, not the development of new phylogenetic algorithms.
Revision made:
- Clearly stated in:
    - **Abstract**
    - **Conclusion**
    - **Section: Scope and Intended Usage**
- Replaced strong claims like *"methodological advantage"* with:

© 2026. Published by The Company of Biologists under the terms of the Creative Commons Attribution License (https://creativecommons.org/licenses/by/4.0/).

    - “workflow enhancement”
    - “integration framework”
    - “comparative analysis platform”

Comment 2: Suggestion to include Parsimony and Bayesian methods
Response:
- We have **successfully integrated Parsimony (MP)** using PHYLIP DNAPARS.
- Bayesian methods are acknowledged as important but left as future work.

Revision made:
- Added Parsimony in:
    - **Methodology → Tree Inference Methods**
    - **Design Chapter → Parsimony Implementation Section**
- Mentioned Bayesian inference in:
    - **Scope and Limitations**
    - **Future Work Section**

Comment 3: Clarify scope and limitations
Response:
We have explicitly clarified that the pipeline is:
- A **framework for automation and comparison**
- Not a replacement for specialized phylogenetic tools

Revision made:
- Fully revised:
    - **Scope and Intended Usage section**
- Added:
    - Limitations (no Bayesian, not ultra-large phylogenomics optimized)

Comment 4: Validation is limited
Response:
We expanded validation by:
- Using **5 biologically diverse datasets**
- Including **large dataset experiments (up to 200 species)**

Revision made:
- Updated:
    - **Dataset Introduction (added large dataset experiment)**
    - **Results and Analysis section**
- Clearly stated scalability evaluation

Comment 5: Avoid “methodological advantage”
Response:
All such terms have been removed or rephrased.
Revision made:
- Updated throughout manuscript:
    - Abstract
    - Results
    - Conclusion

Comment 6: Reference errors
Response:
All reference issues have been corrected.
Fixes:
- Corrected author names ([9])
- Fixed journal abbreviation ([21])
- Corrected spelling issue ([31])
- Removed/replaced missing reference [17]

Response to Reviewer 2
Comment 1: Missing alignment data → reproducibility issue
Response:
We fully agree. To improve reproducibility:
Revision made:
- Added:
    - **All alignment files**
    - **Datasets used in experiments**
- Provided:

© 2026. Published by The Company of Biologists under the terms of the Creative Commons Attribution License (https://creativecommons.org/licenses/by/4.0/).

    - **GitHub repository link containing:**
        - Code
        - Datasets
        - Alignment outputs

Where added:

- Section: **Code and Data Availability**

Comment 2: Target audience not clearly defined

Response:

We have now explicitly defined the target users.

Revision made:

Added in:

- **Scope and Intended Usage section**

Includes:

- Graduate students
- Bioinformaticians
- Researchers using command-line tools

Comment 3: Comparison with Galaxy

Response:

We added a clear comparison with Galaxy.

Revision made:

- Included explanation in:
    - **Scope and Intended Usage**
- Added citation for Galaxy platform
- Highlighted:
    - GUI vs command-line difference
    - Flexibility vs ease-of-use trade-off

Comment 4: Can pipeline handle concatenated alignments?

Response:

- Clarified that pipeline currently supports **single-gene datasets**
- Mentioned multi-gene concatenation as **future work**

Revision made:

- Added in:
    - **Scope**
    - **Future Work**

Comment 5: Minor language and formatting issues

Response:

All minor issues have been corrected:

Fixes include:

- Section titles corrected
- Sentences reworded for clarity
- Species names italicized
- Ambiguous sentences rewritten

<u>Second decision letter</u>

MS ID#: bio.062482R1

MS Title: A Python-Based Automated Pipeline for Phylogenetic Tree Construction, Visualization, and Comparative Statistical Evaluation

Authors: Bhavika Gambhava, Janvi Patel and Niyati Buch

I have now reached a decision on the above manuscript.

The reviewer reports are shown at the bottom of this email.

© 2026. Published by The Company of Biologists under the terms of the Creative Commons Attribution License (https://creativecommons.org/licenses/by/4.0/).

As you will see, there are still very minor points that require your attention. Please address them in a revised version.

At this stage, we also ask you to ensure your manuscript complies with our formatting guidelines – please see our manuscript preparation guidelines for details. Provided you are able to fully address the referees' comments, we are positive about publication of your paper (we accept over 95% of revision submissions) and therefore hope you won't mind any extra work involved in reformatting your manuscript at this point.

Please upload both a 'clean' version of your Word file, along with a highlighted version clearly showing where you have made changes in the revised manuscript. Please avoid using 'Track changes' in Word files as these are lost in PDF conversion.

I should be grateful if you would also provide a point-by-point response detailing how you have dealt with the points raised by the reviewers in the 'Response to Reviewers' box. Please attend to all of the reviewers' comments. If you do not agree with any of their criticisms or suggestions please explain clearly why this is so.

Reviewer 1

Comments for the author

I am satisfied with this revision. Hope that this paper can be published soon.

Reviewer 2

Comments for the author

Many of the reviewer comments have now been addressed sufficiently. However, https://github.com/JanviJPatel30/PhylogeneticPipeline still does not contain the datasets and outputs. Therefore I have once again selected no for the reproducibility questions.

Minor comments on newly added text:
- Page 6 line 6: mentions the inclusion of parsimony in a way that seems to reference the reviewer comments. When introducing to the reader, it should mention parsimony and likelihood.
- Page 6 line 40: this pipeline isn't necessarily any more flexible than those offered by Galaxy. However, it does offer the statistical comparisons.
- When listing the datasets on page 19, please add the number and length of sequences.
- The final sentence, "The current framework thus provides a strong foundation for the development of next-generation intelligent phylogenetic systems that combine evolutionary bioinformatics with modern computational intelligence techniques" is overblown. It's not clear what you are trying to say, but if you suggest that a user will use LLMs or AI agents to build pipelines, they will probably bypass using this script.

Reviewer's Responses to Questions

Experimental quality
Are experiments performed using appropriate methods that will answer the question (or test the hypothesis or support the observations) posed by the authors? Is the right tool used for the job?
If 'No', please indicate reasons in Comments for Author box below.
Reviewer #1:
- Yes

Reviewer #2:
- Yes

Were the data analyzed using appropriate statistical tests?
If 'No', please indicate reasons in Comments for Author box below.

© 2026. Published by The Company of Biologists under the terms of the Creative Commons Attribution License (https://creativecommons.org/licenses/by/4.0/).

Reviewer #1:
- Yes

Reviewer #2:
- Yes

Reproducibility
Were experiments performed using adequate number of biological replicates?
If 'No', please indicate reasons in Comments for Author box below.
Reviewer #1:
- Yes

Reviewer #2:
- No

Is there sufficient raw data to assess the rigor of the analysis?
If 'No', please indicate reasons in Comments for Author box below.
Reviewer #1:
- Yes

Reviewer #2:
- No

Does the methods section provide sufficient detail to permit reproducibility?
If 'No', please indicate reasons in Comments for Author box below.
Reviewer #1:
- Yes

Reviewer #2:
- Yes

Completeness
Are the manuscript's conclusions supported by the data?
If 'No', please indicate reasons in Comments for Author box below.
Reviewer #1:
- Yes

Reviewer #2:
- Yes

Scholarship
Do the authors cite and discuss the merits of data that would argue for and against their conclusion?
If 'No', please indicate reasons in Comments for Author box below.
Reviewer #1:
- Yes

Reviewer #2:
- Yes

Does the manuscript title & abstract accurately reflect the contents of the manuscript, without hyperbole?
If 'No', please indicate reasons in Comments for Author box below.

© 2026. Published by The Company of Biologists under the terms of the Creative Commons Attribution License (https://creativecommons.org/licenses/by/4.0/).

Reviewer #1:

- Yes

Reviewer #2:

- Yes

Second revision

Author response to reviewers' comments

Response to Reviewer 1

Sr. No.	Comment
1.	I am satisfied with this revision. Hope that this paper can be published soon.
ANSWER	We are grateful to the reviewer for their review stating satisfaction with our revision. Their previous comments were helpful in improving our manuscript.

Response to Reviewer 2

Sr. No.	Comment
1.	Many of the reviewer comments have now been addressed sufficiently. However, https://github.com/JanviJPatel30/PhylogeneticPipeline still does not contain the datasets and outputs. Therefore I have once again selected no for the reproducibility questions.
ANSWER	To comply with this comment, we request the editor and reviewer to kindly note change in the github link. We have put source code, datasets, and outputs to support reproducibility on https://github.com/niyatibuch/automated-phylogenetic-pipeline We have mention the same link in the paper.
2.	Page 6 line 6: mentions the inclusion of parsimony in a way that seems to reference the reviewer comments. When introducing to the reader, it should mention parsimony and likelihood.
ANSWER	We have re-written the "Tree Inference Methods" to comply with this suggestion. We have rephrased a statement in the abstract, also.
3	Page 6 line 40: this pipeline isn't necessarily any more flexible than those offered by Galaxy. However, it does offer the statistical comparisons.
ANSWER	We have re-written the statements according the reviewer's suggestion.
4	When listing the datasets on page 19, please add the number and length of sequences
ANSWER	We have added the details on page 19 as well as 20.
5	The final sentence, "The current framework thus provides a strong foundation for the development of next-generation intelligent phylogenetic systems that combine evolutionary bioinformatics with modern computational intelligence techniques" is overblown. It's not clear what you are trying to say, but if you suggest that a user will use LLMs or AI agents to build pipelines, they will probably bypass using this script.
ANSWER	We have rephrased the sentence according to the reviewer's suggestion.
	We are thankful to the reviewer for the constructive comments and suggestions, which have helped us improve the quality, clarity, and reproducibility of the manuscript.

Third decision letter

© 2026. Published by The Company of Biologists under the terms of the Creative Commons Attribution License (https://creativecommons.org/licenses/by/4.0/).

MS ID#: bio.062482R2

MS Title: A Python-Based Automated Pipeline for Phylogenetic Tree Construction, Visualization, and Comparative Statistical Evaluation

Authors: Bhavika Gambhava, Janvi Patel and Niyati Buch

I am happy to tell you that your manuscript has been accepted for publication in Biology Open, pending our standard publication integrity checks. It was accepted on 30th June 2026.

© 2026. Published by The Company of Biologists under the terms of the Creative Commons Attribution License (https://creativecommons.org/licenses/by/4.0/).
